# The Link between Peer Relations, Prosocial Behavior, and ODD/ADHD Symptoms in 7–9-Year-Old Children

**DOI:** 10.1155/2013/319874

**Published:** 2012-12-18

**Authors:** Muirne C. S. Paap, Ira R. Haraldsen, Kyrre Breivik, Phillipa R. Butcher, Frøydis M. Hellem, Kjell M. Stormark

**Affiliations:** ^1^Department of Research Methodology, Measurement, and Data Analysis, Faculty of Behavioural Sciences, University of Twente (Building Chalet), Drienerlolaan 5, 7522 NB Enschede, The Netherlands; ^2^Department of Neuropsychiatry and Psychosomatic Medicine, Oslo University Hospital, Oslo, Norway; ^3^Regional Centre for Child and Youth Mental Health and Child Welfare, Uni Health, Uni Research, Krinkelkroken 1, Bergen, Norway; ^4^Department of Psychology, The Australian National University, Building 39, Canberra, Australia; ^5^Department of Clinical Psychology, University of Bergen, Christies gt 12, 5015 Bergen, Norway

## Abstract

Oppositional defiant disorder (ODD) and attention-deficit/hyperactivity disorder (ADHD) are characterized by symptoms that hinder successful positive interaction with peers. The main goal of this study was to examine if the presence of symptoms of ODD and ADHD affects the relationship between positive social behavior and peer status found in 7–9-year-old children who show symptoms typical of ADHD and/or ODD. Furthermore, the possible interaction with sex was investigated. We used data collected in the first wave of The Bergen Child Study of mental health (BCS), a prospective longitudinal total population study of children's developmental and mental health. The target population consisted of children in the second to the fourth, in all public, private, and special schools in Bergen, Norway, in the fall of 2002 (*N* = 9430). All 79 primary schools in Bergen participated in the study. Both teacher (8809 complete cases) and parent (6253 complete cases) report were used in the analyses. ADHD and ODD scores were estimated using the Swanson Noland and Pelham rating scale version IV (SNAP-IV), and peer problems and prosocial behavior were assessed using the Strengths and Difficulties Questionnaire (SDQ). We replicated the relationship between peer problems and prosocial behavior found previously in typically developing children. Our results showed that the relationship between peer problems and prosocial behavior became weaker as the ODD symptoms increased in number and severity. For ADHD this effect was only found in the teacher report of the children. A sex effect for ODD symptoms was found only using the parent report: boys with ODD symptoms showed less prosocial behavior than girls with similar levels of ODD symptoms. Since this effect was not found using the teacher data, it may imply a situational effect (school/home) for girls with high levels of ODD. The moderator effect of ODD/ADHD was comparable for boys and girls. Our findings suggest that even if children with ADHD/ODD symptoms have the opportunity to practice their social skills in peer relationships, this is not necessarily accompanied by an increase in prosocial behavior.

## 1. Introduction

Oppositional defiant disorder (ODD) and attention-deficit/hyperactivity disorder (ADHD) are characterized by symptoms that, by their very nature, are likely to hinder successful positive interaction with peers (see [1]). While negativistic, defiant, disobedient, and hostile behavior toward authority figures is central to the definition of ODD [2], other characteristics such as the tendency to blame or deliberately annoy others, and to be angry or resentful, can also be directed against peers (see [3]). Reported prevalence rates for ODD in children range from 2 to 17% [4–6], with a cross-cultural estimated prevalence of 3.3% [7]and a lifetime estimated prevalence of 10.2% [8]. ADHD is characterized by a persistent pattern of inattention and/or hyperactivity-impulsivity that is both maladaptive and inappropriate for the developmental level [2]. Reported prevalence rates of ADHD range between 1 and 17% of all school-aged children [9–11], with a European estimated prevalence of 5% [12] and a worldwide estimated prevalence of 5.29% [13]. In many children, ODD is accompanied by ADHD. 

Unsurprisingly, difficulties with interacting positively and constructively with peers are frequently reported in children with ODD and/or ADHD [14–17]. Children with ADHD, for example, show high rates of negative and uncooperative behaviors [14, 18, 19], and their behavior is generally insensitive and disruptive in various social contexts [1]; while ODD is associated with increased hostility towards, and decreased resistance to, provocation by peers [15]. 

 These findings suggest that children with ODD and/or ADHD may be at a serious disadvantage with regard to their social development. Research has shown that positive social skills such as friendly and cooperative behavior, and prosocial behaviors such as giving emotional support, showing empathy and sharing, allow children to succeed on social tasks and are associated with peer popularity; children who demonstrate poor social skills, disruptive, physically aggressive, and antisocial behavior, however, have been shown to be at risk of experiencing peer rejection [20–24]. Further, the association between peer relationships and positive social behaviors is thought to be reciprocal: positive social behaviors foster good peer relationships and good peer relationships foster positive social behaviors [25]. In this study, we will investigate whether we can replicate the relationship found between peer status and prosocial behavior in a population-based study (see hypothesis 1 below). If these associations hold for children with ODD and/or ADHD, then their social development is likely to be at risk: their poor social skills are likely to impair relationships with peers, and this will in turn reduce their opportunity to practice and develop positive social behaviors.

However, it is not yet clear how peer relationships and social behavior are associated in children with ODD and/or ADHD. While well-liked typically developing children combine high levels of positive social behaviors with low levels of negative social behaviors, children with ADHD may, for example, show high levels of both positive and negative social behavior. Research suggests that children with ADHD are not unwilling to or at a loss how to behave in a prosocial manner. Blachman and Hinshaw [14], for example, found no differences in prosocial behaviors between girls with or without ADHD, even though the girls with ADHD did suffer from social difficulties (fewer friendships, more conflict, and relational aggression). This finding suggests that the relationship between prosocial behavior and peer status is different in children with and without ADHD; in spite of normal levels of prosocial behavior, their negative social behaviors prevent them from being well-liked by peers. However, a study by Mrug et al. [1] found that the peer status of children with ADHD was predicted by the same behaviors as the peer status of typically developing children. Children with ADHD were not well liked if they displayed negative behavior; peer acceptance, on the other hand, was associated with low levels of negative social behavior as well as high levels of prosocial behavior. Given the importance of positive peer relationships for social development, the first goal of our study is to determine if the presence of symptoms of ODD and ADHD affect the relationship between positive social behavior and peer status (see hypothesis 2 below). 

 According to the DSM-IV-TR, ODD is more prevalent in boys than girls before puberty [2]. ADHD is also found to be more common in boys: they are 2.5–5.6 times more likely to be diagnosed with ADHD than girls [26]. As a consequence, most studies that report on ODD and ADHD pertain to boys. Nevertheless, there are some exceptions, and these have shown highly interesting results. First, girls with ADHD suffer even greater levels of peer rejection than their male counterparts [27, 28], even though they were found to display more prosocial behavior than boys in a recent study [29] and show normal levels of prosocial behavior compared to girls without ADHD [14]. A recent review of peer problems in children with ADHD pointed out that several studies have found sex differences regarding the *type* of negative social behavior shown by children with ADHD [30]. More specifically, boys were found to show higher rates of interrupting, leaving their seats, aggression, and more severe rule breaking behaviors [31]. While these behaviors are not necessarily directed at peers, they have been shown to correlate with peer rejection [1]. Girls with ADHD were more likely to engage in verbal aggression towards peers and to use relational forms of aggression, such as spreading rumors and excluding others [31–33]. This could suggest that the type of negative social behavior of girls with ADHD (and/or ODD) may be more detrimental to peer interactions than that of boys. Summarizing, research findings suggest that girls with ADHD show more prosocial behavior than boys, but suffer more peer rejection. Hence, the relationship between prosocial behavior and peer status could be expected to be weaker for girls with ADHD than for boys. In this study, we want to test this by investigating whether the effect of ODD and ADHD symptoms on the relationship between positive social behavior and peer status is different for boys and girls (see hypothesis 3 below).

The purpose of this study was to determine whether the relationship between peer problems and prosocial behavior found in children who show symptoms typical of ODD and/or ADHD is similar to the one found in typically developing children. We will test three hypotheses.

1. We expect to replicate the relationship between peer relations and prosocial behavior in typically developing children, in a population based study.
2. The more ODD/ADHD symptoms a child displays, the weaker the relationship between peer problems and prosocial behavior (moderator effect of ODD/ADHD).
3. We expect that the moderating effect of ODD/ADHD symptoms is larger for girls than for boys.

## 2. Method

### 2.1. Subjects

The current study uses data collected in the first wave of The Bergen Child Study of mental health (BCS), an ongoing prospective longitudinal total population study of children's developmental and mental health. The main aim of the BCS is to establish reliable prevalence data for mental health problems in children in a total population. For more information about the BCS we refer to Heiervang et al. [34]. The study was approved by the Regional Ethics Committee on Medical Research.

The target population consisted of children in the second to the fourth grade in all public, private, and special schools in Bergen, Norway, in the fall of 2002 (*N* = 9430). Bergen is the second largest city of Norway, with a total population of around 235,000. All 79 primary schools in Bergen participated in the study. In the first stage of the BCS, a four-page screening instrument was completed by both teachers and parents. The screening instrument was sent to the teachers, together with a letter explaining the purpose of the study and an informed consent form. The teachers were asked to pass on an extra copy to all the parents of children in their class; the parents of 7007 children agreed to participate and returned a signed informed consent form. As a special feature of the study, teachers returned completed questionnaires even when the informed consent form was not returned by the parents, but without any personal identification, to ensure that these questionnaires could not be traced to individual children [35, 36]. 

Separate datasets were generated for teacher and parent data; listwise deletion of cases with missing values for sex, grade, Swanson Noland and Pelham rating scale version IV (SNAP) items, or Strengths and Difficulties Questionnaire (SDQ) items was implemented in each separate file. The parent file contained 6253 complete cases (50% boys; 33% grade 2, 35% grade 3, 32% grade 4) and the teacher file contained 8809 complete cases (51% boys; 33% grade 2, 34% grade 3, 33% grade 4). 

### 2.2. Measures

The four-paged screening instrument included the Strengths and Difficulties Questionnaire (SDQ), a brief multidimensional measure of psychological adjustment of children aged 3 to 16 years [37, 38], and the Swanson Noland and Pelham rating scale version IV (SNAP-IV), a screening instrument for ADHD and ODD based on the DSM symptom lists [39]. 

The SDQ consists of 25 items that are scored on a 3-point scale (0 = “not true,” 1 = “somewhat true,” and 2 = “certainly true”). The 25 items are meant to cover five dimensions, each consisting of five items, generating scores for the following: hyperactivity, emotional symptoms, conduct problems, peer problems, and prosocial behavior. In our study, we used the latter two. The questionnaire can be completed by the parents and/or teachers of 4- to 16-year-olds. The SDQ is available in over 40 languages [40] and is being widely used in epidemiological, developmental, and clinical research [41]. A recent factor-analytic study of BCS data demonstrated that the subscales PRO (prosocial behavior) and PPR (peer problems) are valid scales: the items in these subscales showed high loadings (0.6–0.8) on their own subscale, and low loadings (0–0.2) on the other subscales [35]. Therefore, we deem these scales “safe for use” in this study.

The SNAP-IV comprises 26 items divided over three subscales: Inattentiveness (nine items), Hyperactivity/Impulsivity (nine items), and Oppositional behavior (eight items). On the original form, the items are scored on a 4-point scale (0 = not at all, 1 = just a little, 2 = much, and 3 = very much) [39]. In the Bergen Child Study, three-point scales were used to provide identical response categories for the entire questionnaire (0 = “not true,” 1 = “somewhat true,” and 2 = “certainly true”) [42]. The current study used the subscales Inattentiveness and Hyperactivity/Impulsivity to measure ADHD and “Oppositional behavior” to measure ODD. The SNAP-IV is frequently used in screening for ADHD and assessing clinical interventions. Its internal consistency has been found to be good to excellent [43]. A recent study of the psychometric properties of the Chinese SNAP-IV showed adequate test-retest stability and validity [44]. Several recent studies of ADHD inventories have found support for the presence of one strong general ADHD factor [45–47]. We confirmed this finding using Mokken Scale Analysis (MSA), a form of nonparametric Item Response Theory (IRT). Therefore, we chose to use the total score on *all* ADHD items as an indicator for ADHD in this study. IRT is becoming increasingly popular in psychiatric research, both for analyzing the dimensional structure of questionnaires [48–50], as well as scrutinizing formal diagnoses (see e.g., [51, 52]). A discussion of MSA is beyond the scope of this paper; we refer the interested reader to Sijtsma and Molenaar [53].

### 2.3. Statistics

Separate regression analyses for parents and teachers were used to determine whether peer problems predicted (here, “predict” is used as a mathematical term only and no causal relationship is implied) prosocial behavior. We standardized all variables prior to the regression analyses. This allowed us to determine whether the linear regression models based on parent and teacher data, respectively, were similar. Prosocial behavior served as a dependent variable, and ADHD, ODD, sex (boy = 1, girl = 0), and grade (dummy coded; reference category = grade 4) served as independent variables. To test whether a high number of ADHD/ODD symptoms moderated the effect between peer problems and prosocial behavior, we included interaction effects in our models. We also tested whether there was a significant interaction between sex and ADHD/ODD. The models were built using a forward procedure, adding one variable at a time and checking whether it was significant; but also monitoring its potential impact on the size and *P* value of the previously entered variables, as well as multicollinearity diagnostics. We started with the control variables sex and grade, and continued by adding peer problems, ADHD, ODD, and the moderator effects (peer problems × ADHD/ODD). All regression analyses were performed in SPSS 16 [54].

## 3. Results

### 3.1. Descriptive Statistics

Table 1 shows descriptive statistics for prosocial behavior, peer problems, and the 3 SNAP-IV scales. To allow comparison of the subscales, all reported scale scores are average scores (not sum scores); it follows that the theoretical maximum for all subscales is equal to “2”. Table 1 shows that girls were significantly more prosocial and had less peer problems, ADHD, and ODD symptoms than boys. These findings were similar for teacher and parent report. Interestingly, a striking difference in means was found for ODD: a mean of 0.22 was found for girls using parent report, compared to 0.08 based on the teacher report. Note that for all comparisons listed in Table 1, *P* < 0.001. 

### 3.2. Hypothesis Testing: Linear Regression Analyses

Multicollinearity was only a problem when both moderator effects were entered in the same model. Therefore, *two* final models were estimated (see Table 2), one including the moderator effect of ADHD (model 1), and one including that of ODD (model 2). Interaction effects were not included in the final model if they were not significant.

The effect of sex and grade was similar in size for the parent and teacher data-sets. Boys displayed significantly less prosocial behavior than girls (*P* < 0.001 for both parent and teacher report). Children in second grade showed somewhat less prosocial behavior than children in fourth grade, this effect was significant (*P* = 0.002 and *P* = 0.003, for teacher and parent report, resp.). The difference between third and fourth graders was not significant (*P* = 0.499 and *P* = 0.267, for teacher and parent report, resp.). Although the effect of peer problems was significant for both data-sets (*P* < 0.001 for both parent and teacher report), it was about twice as large when based on the teacher data. The same applies to the effect of ADHD-score (*P* < 0.001 for both parent and teacher report). The effect of ODD was also significant, and larger than the effect of ADHD (twice as large based on the teacher data, almost four times as large based on the parent data; *P* < 0.001 for both parent and teacher report). Thus, an increase in ADHD or ODD symptoms was accompanied by a decrease in prosocial behavior. The moderator effect of ADHD was only significant for the teacher data; children with higher levels of ADHD showed a weaker relationship between peer problems and prosocial behavior (*P* < 0.001). The moderator effect of ODD was significant in both data-sets (*P* < 0.001 for both parent and teacher report). In the parent data-set, an interaction was found between sex and ODD; a stronger relationship between prosocial behavior and ODD symptoms was found for boys compared to girls (*P* = 0.003).

**Figure 1:**
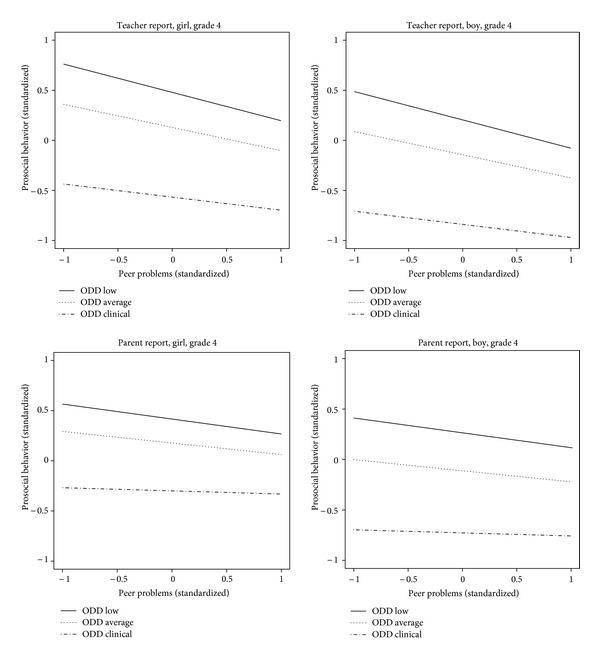
Graphical illustration of the moderator effect of ODD on the relationship between peer problems (*x*-axis) and prosocial behavior (*y*-axis). Different plots were made for teacher and parent report, as well as for girls and boys. The effects shown are those found for children in grade 4.

A graphical illustration of the moderator effect of ODD can be found in Figure 1. Note that all variables were standardized. Thus, a value of “0” corresponds to an average level. The relationship between peer problems and prosocial behavior is depicted for three relevant levels of reported ODD symptoms: virtually none (corresponding to the value −1), average (value 0) and a score above the 95th percentile (which is considered the clinical cut-off, corresponding to a value of approximately 2 in our data; [55]). As can be seen from the slope of the lines in Figure 1, the relationship between peer problems and prosocial behavior is weaker for children with a clinical level of reported ODD symptoms compared to the other two groups. It can also be observed that the slopes based on the parent report are weaker for all groups. A striking difference is seen between girls and boys based on the parent report: if ODD symptoms are absent, there is no gender difference, but if the parents report many ODD symptoms, they observe much less frequent prosocial behavior in boys than in girls. Teachers also report less prosocial behavior in boys, but this difference is stable among the three groups.

## 4. Discussion

As expected, we replicated the relationship between peer problems and prosocial behavior found in typically developing children. We hypothesized that ADHD and ODD would act as moderator variables, weakening the relationship between peer problems and prosocial behavior. Consistent with this expectation, we found that the relationship between peer problems and prosocial behavior became weaker as the ODD symptoms increased in number and severity. For ADHD this effect was only found in the teacher report of the children. A sex effect for ODD symptoms was found only using the parent report: boys with ODD symptoms showed less prosocial behavior than girls with similar levels of ODD symptoms. We did not find support for our third hypothesis: the moderator effect of ODD/ADHD was comparable for boys and girls.

 Previous studies have shown a relationship between peer status and prosocial behavior in various age-groups and countries. Our findings provide further support for this relationship in 7–9-year-old children. Importantly, we found a relationship between peer problems and prosocial behavior, even when other variables were taken into account (such as sex and ADHD/ODD). Since we used a very large (*N* = 9430) population-based study, we feel confident about the generalizability of these findings. As noted by Veenstra et al. [22], it is highly important to investigate these issues in young children so that it can be predicted which children are likely to develop problematic behavior later on and which children are unlikely to do so. Both parents and teachers can play an important role in timely identification of problems, facilitating both prevention and treatment schemes. 

 Collectively, our findings suggest that symptoms that are associated with attention-deficit and disruptive behavior disorders moderate the relationship between peer problems and prosocial behavior. More specifically, children reported to display clinical levels (>95th percentile) of ADHD/ODD show very little prosocial behavior, regardless of whether or not they are reported to have problems with peers. As mentioned before, the relationship between peer status and prosocial behavior is thought to be reciprocal; however, our findings may imply that this is not necessarily true for children with high levels of ADHD/ODD symptoms. Even those children with a high level of reported ADHD/ODD symptoms that have *good* peer relationships, and thus would have ample opportunity to practice and develop prosocial behavior skills, had a low predicted prosocial score. A potential explanation could be that (a subgroup of) these children tend to overestimate their own social competence more than typically developing children do [56]. Indeed, a recent study showed that children with ADHD who had positively biased self-perceptions showed less prosocial behavior than other children with ADHD as well as controls [57]. Linnea et al. suggest that poor social skills in children with ADHD may be due to less skillful social behavior preventing them to accurately assess and adjust their behavior, and not so much due to the ADHD symptoms. This may explain, in part, why Blachman and Hinshaw [14] did not report any differences in positive social behaviors between girls with and without ADHD. They investigated patterns of friendship in girls with clinically diagnosed ADHD compared to typically developing girls, who attended a 5-week naturalistic summer camp. The ratings Blachman and Hinshaw used were based on self-report, and only reports from girls that had at least developed one friendship during the summer camp were included. As mentioned previously, self-perceptions may be positively biased in children with ADHD, so this may explain why Blachman and Hinshaw did not find any differences between girls with and without ADHD. 

Although not the main focus of our study, the finding that girls had a higher prosocial score than boys warrants some discussion. This finding in favor of girls is quite consistent in the literature [23, 58–61]. Hastings et al. [59] provide several possible explanations for this finding, some of which will be presented here. Firstly, they note that many studies employ parent and teacher report. However, studies using other methods, such as observational studies, indicate a smaller sex difference in prosocial behavior. Therefore, Hastings et al. reason that apparent sex differences in prosocial behavior may be linked to a culturally related belief that girls “are made of everything nice,” to which parents and teachers are more prone than trained observers. In other words, the sex difference may be an effect of sex-typical socialization. Furthermore, Hastings et al. pose that gender may serve as a multi-faceted variable: a “summary” variable that encompasses biological predispositions, sex-typical socialization from parents and peers, media, and so on. Finally, they suggest that the definition of prosocial behavior used in research may be too narrow: perhaps it predominantly reflects positive behavior typically displayed by girls/women, whereas males might use a distinct set of prosocial behaviors that may have been overlooked. As for the first two points: it has been proposed that gender differences in general might be smaller in the Scandinavian countries, men and women being more equal than in other societies (see [62]), which would translate into weaker stereotypes about sex differences. As a consequence, Scandinavian girls and women might not be subjected to stereotype threat as frequently as females from other countries. Therefore, we expect that culturally held beliefs may not have had such a big impact on the way that Norwegian parents and teachers rated the children's behavior in our study. This is an assumption which needs to be formally investigated in a future study. When it comes to the definition of prosocial behavior in research, we cannot rule out that the used subscale may reflect positive behaviors more commonly displayed by females than by males. More research in this area is needed before any firm conclusions can be drawn.

In this study, we used both parent and teacher ratings. It is important to note that informants tend to differ in their perceptions of and interactions with children, and that these take place in different situations which may provoke different types of behavior in children. As noted by Veenstra et al. [22], a highly relevant difference between teachers and parents is their ability to make comparative judgments: where teachers are in the position to observe a whole range of positive and negative behaviors among their pupils, parents have deeper knowledge of their children's behavior in a wider range of settings. It is only to be expected that these differences translate into different scores on teacher versus parent report. We suggest that these differences should not be regarded as “bias,” but that the teacher and parent are both valuable sources of information, each providing a “piece of the puzzle.” This belief is further bolstered by findings of studies examining the sensitivity of the SDQ; it has been shown that the greatest sensitivity in community and clinical samples in detecting any psychiatric disorder using teacher and parent ratings was obtained when these ratings were combined [63, 64]. Many other studies have shown rater differences either with respect to reported levels of ADHD, ODD or social behavior, or the outcome of the analyses [65–72]. Therefore, it came as no surprise that there were some differences between parents and teacher ratings in our study as well. In our opinion, this underlines the importance of including both parent and teacher ratings when studying aspects of the child's social behavior. 

The most important differences between teacher and parent report were as follows: the weaker overall relationship between prosocial behavior and peer problems, the absence of the moderating effect of ADHD in the parent data, and the presence of an interaction of ODD symptoms and sex in the parent data. Currently, we can only speculate about possible explanations. We expect that teachers have ample opportunity to observe a relationship between the child's conduct (both related to ADHD/ODD and positive social interaction) and peer status. However, if children do not have a lot of friends (possibly due to conduct problems), parents may not be in the position to judge the relationship between peer problems and prosocial behavior very well. Not only would there be little opportunity to observe social interactions, but the diversity of these behaviors may also be limited if the child only has one or two friends. This may explain the weaker relationship between peer status and prosocial behavior reported by parents. On the other hand, parents have the opportunity to observe their child's behavior outside of the class-room setting. Maybe girls with high levels of ODD symptoms act out more at school when interacting with a peer-group that may be partly hostile towards them, but show more prosocial behavior at home when interacting with their friend(s) or siblings. In a recent study, Mikami and Lorenzi [73] found that girls with comorbid conduct problems were more likely to show peer problems relative to boys with similar levels of conduct problems. If we take our findings and those of Mikami and Lorenzi together, and if we assume that the parent-reported findings reflect a situational effect, girls with high levels of ODD and peer problems show more prosocial behavior than (1) boys with similar scores, and (2) they do in the class-room setting, but experience more peer problems than their male counterparts. It follows that this could imply that these girls do possess some basic prosocial skills but choose not to use these in certain settings. A future study is needed to investigate whether girls with ODD show less prosocial behavior at school than at home, as our findings seem to indicate. Preferably, the child's behavior would be observed by the same person both at school and at home, to make sure the effect can be attributed to the situation. 

 Even though the screening measure we used to measure ADHD and ODD directly reflected the symptoms for ADHD and ODD as they are listed in the DSM-IV, we cannot rule out that some of the ODD/ADHD symptoms were actually caused by other disorders. For example, several symptoms of increased arousal in PTSD show an overlap with ADHD (and ODD) symptoms, such as sleeplessness, irritability, or anger, difficulty concentrating, hypervigilance, and exaggerated startle response [74]. We concur with Weinstein et al. [74], that it could be beneficial to include items that assess situational factors related to hyperactivity/impulsivity and oppositional behavior, such as feared places or people, in order to elucidate whether the ODD/ADHD items are in fact more indicative of PTSD.

In summary, we replicated earlier findings that girls show more prosocial behavior than boys, and that peer relations and prosocial behavior are correlated. Moreover, we found support for our hypothesis that symptoms indicative of ADHD (teacher report only) and ODD moderate (weaken) the relationship between peer relations and prosocial behavior. Since prosocial behavior has been found to be a key predictor for future social adjustment (e.g., [75]), it is very important to address poor (pro)social skills at an early stage. Several studies have shown that interventions such as Social Skills Training (SST) can have a positive effect on the social interactions of children with ADHD [76, 77]. In these training programs, the focus is often on strengthening the child's prosocial skills (e.g., [76]). However, our findings suggest that even if children with ADHD/ODD symptoms have the opportunity to practice their social skills in peer relationships, this is not necessarily accompanied by an increase in prosocial behavior. Taken together with the findings reported by Linnea et al. [57] that poor social skills in children with ADHD may be due to less skillful social behavior preventing them to accurately assess and adjust their behavior, we suggest that treatment programs for children with ADHD/ODD should focus on promoting accurate self-perception of social skills in these children, in addition to practicing prosocial skills. Research has shown that girls with conduct problems are at a larger social disadvantage than their male counterparts [73]. If girls with ODD indeed choose to show less prosocial behavior in the classroom than they do at home as our findings seem to suggest, this may be a result of the school climate. If this is the case, a school-based intervention might be warranted, including anti-bullying interventions.

## Figures and Tables

**Table 1:** Sex comparisons of the means (SD) for the variables prosocial behavior, peer problems, ADHD, and ODD.

	Girls	Boys	*t *
Teacher data			
* N *	4341	4468	
Prosocial behavior	1.78 (.33)	1.56 (.46)	25.17*
Peer problems	.13 (.26)	.20 (.34)	−9.85*
ADHD	.16 (.23)	.26 (.32)	−14.72*
ODD	.08 (.20)	.20 (.37)	−21.00*
Parent data			
* N *	3126	3127	
Prosocial behavior	1.76 (.27)	1.65 (.32)	15.48*
Peer problems	.17 (.28)	.21 (.32)	−4.95*
ADHD	.09 (.19)	.25 (.35)	−26.56*
ODD	.22 (.29)	.29 (.36)	−8.52*

^∗^Statistically significant at **α** = 0.05.

**Table 2:** Results of the regression analyses with prosocial behavior as the dependent variable.

	Teacher data						Parent data					
	Model 1			Model 2			Model 1			Model 2		
	**β**	SE	*t *	**β**	SE	*t *	**β**	SE	*t *	**β**	SE	*t *
Intercept^a^	.14	.018		.13	.018		.18	.024		.18	.024	
Sex^b^	−.27*	.018	−14.92	−.27*	.018	−14.91	−.29*	.024	−12.30	−.29*	.023	−12.40
Grade 2	−.07*	.022	−3.07	−.07*	.022	−3.02	−.09*	.029	−2.97	−.08*	.029	−2.94
Grade 3	.02	.021	.68	.02	.021	.73	−.03	.028	−1.04	−.03	.028	−1.11
ppr	−.22*	.011	−20.19	−.23*	.011	−21.42	−.09*	.014	−6.79	−.11*	.014	−7.78
ADHD	−.14*	.013	−10.50	−.12*	.012	−9.64	−.07*	.017	−4.76	−.07*	.016	−4.65
ODD	−.31*	.012	−26.22	−.35*	.013	−27.05	−.27*	.015	−17.77	−.24*	.020	−11.93
ppr × ADHD	.03*	.006	4.95	—	—	—	n.s.			—	—	—
ppr × ODD	—	—	—	.05*	.006	8.13	—	—	—	.04*	.008	4.23
ODD × sex	—	—	—	n.s.			—	—	—	−.07*	.024	−2.93

*Note*: all continuous variables were standardized prior to the regression analyses. ADHD: attention-deficit/hyperactivity disorder; ODD: oppositional defiant disorder; ppr: peer problems. ADHD and ODD scores were measured with the corresponding subscales from the Swanson Noland and Pelham rating scale version IV. Prosocial behavior (dependent variable) and ppr were measured with the corresponding subscales from the Strengths and Difficulties Questionnaire.

*Statistically significant at **α** = 0.05, ^a^ *t*-values are not reported because testing whether the intercept is different from 0 is irrelevant in our study, ^b^1 = boy, 0 = girl.
